# 

**Published:** 1879-06

**Authors:** 


### INDEX TO VOLUME XXXVIII.

PAGE.

### A.

Abscess, retro-œsophageal, F. E. Waxham, M. D. .... 273
Adulteration of sugars, syrup and honey, Dr. Kedzie .... 278
Alcohol:
Antidote for strychnia, M. Hameau .... 626
Physiological action of, M. Rabuteau .... 429
Amniotic fluid, origin of, Dr. Gusserow .... 183
Amber, oil of, in angina, Dr. Finck .... 439
Anæmia Pernicious, alteration of the sympathetic in, Brigidi. 634
Anæsthesia, by mixture of oxygen and protoxide of nitrogen, M. Paul Bert .... 396
Obstetrical, Dr. E. Hervieux. 99
Andrews, Edmund, M. D.:
Is the hypodermic injection of piles dangerous .... 50
A cure of hæmorrhoids by hypodermic syringe .... 476
The inconveniences of the plaster jacket .... 358
Andros, Frederick, M. D., obituary notice of .... 219
Angina:
Pectoris chloroform in .... 512
Oil of amber in, Dr. Finck .. 439
Announcements for months .... 112, 224, 336, 448, 560, 672
Antaphrodisiac, salicylic acid as an, Dr. Jewett .... 107
Antiseptic surgery:
At Halle, Drs. Volkmann and Kraske. .... 23
A. Goldspohn, M. D .... 485
C. T. Parkes, M. D. .... 501
G. B. Pratt, M. D .... 594
Antiseptic treatment of wounds:
Prof. Lefort on, translated by H. M. Lyman, M. D .... 623

PAGE.
With cotton impregnated with camphor and glycerine, M. Paoli .... 431
Anthrax, cupping in, H. Hunt, M. D. .... 363
Anus, artificial:
Dr. F. E. Waxham .... 46
Prolapsus of, treatment of, by Prof. Langenbeck .... 382
Aorta, spentaneous rupture of, H. L. Harrington, M. D. .... 362
Asthma, rapid relief of, by hypodermic injections of morphia, Dr. Huchard .... 106
Atropia, duboisia, eserine, pilocarpine, muscarine, by E. L. Holmes, M. D .... 355
Atropine, some unusual effects of, F. C. Hotz, M. D .... 36
Auricle and auditory canal, eczema of, Dr. Jones' clinic, F. C. Shaefer, M. D. .... 153

### B.

Bacteria, influence of motion on, Dr. B. A. Harrath .... 208
Ballard, L. Anna, M. D., cases of ovariotomy, in service of Dr. Mary H. Thompson .... 600
Barnes, Robert, M. D., Emmet's operation .... 643
Bartlett, John, M. D., placenta prævia .... 159
Battery hint, R. Tilley, M. D... 519
Belfield, W. T., M. D., has the mammalian blood-corpuscle a nucleus .... 261
Bogue, R. G. M. D.:
Intra-laryngeal tumor .... 375
Gangrene of the leg from phlegmasia dolens .... 605
Bridges, V. R., M. D., poisoning by strychnia .... 48
Bursal tumors, treatment of, Prof. Péan .... 73

PAGE.
Butyl chloral, Dr. Liebreich... 328
Byford, Wm. H., M. D., puerperal vaginitis and laceration, etc. 113

### C.

Cæsarian operation, utero-ovarian amputation complementary to, Dr. Debauge 324
Caldwell, W. S.:
Clinical notes from Paris, 71, 283
Surgical notes from Berlin... 378
Cancer of rectum, in which colotomy was performed, J. H. Salisbury, M. D. 270
Cantharidine, in treatment of nævus 555
Carbolic, cresylic acids and creosote, tests for 335
Catarrh nasal, powder for use in, Dr. Robinson 302
Cerebellum, dermoid cyst in the, Dr. Irvine 435
Cerebrum, lesions of the anterior lobes, M. Quenen 397
Cervix uteri:
Laceration of, Dr. W. H. Newman 400
Puerperal laceration of, and trachelorrhaphy as a cure, E. C. Dudley, M. D. 225
Action of tinct. of iodine on, Dr. Laboulbène 327
Cerumen (impacted) and polypus in external meatus, Dr. Jones' clinic, F. C. Shaefer, M. D. 155
Chair, operating, devised by Dr. H. J. Bigelow 511
Chemistry, report on, J. H. Salisbury, M. D. 179
Chenopodium, oil of, fatal poisoning by, T. R. Brown, M. D. 201
Chloral butyl, Leibrich 328
Chloral:
In malignant cholera, Surgeon-Major Hall 430
As a revulsive 556
And morphine in tetanus, M. Broca 552
Cholera, chloral in malignant, Surgeon-Major Hall 430
Chloroform asphyxia, a case of, unusually late, F. C. Hotz, M.D 591
Chloroform, in angina pectoris. 512
Chrysophanic acid in psoriasis. 553
Climatology, F. C. Shaefer, M.D 136

PAGE.
Coccobacteria septica, translated from German of Billroth and Ehlich, by J. W. Dal, M. D. 540
Colotomy, in case of cancer of rectum, J. H. Salisbury, M.D.. 270
Coma and alcohol differentiation, Dr. Macewen 361
Cornea, structure of, Dr. Ranvier 671
Corpuscle, has the mammalian blood, a nucleus, Wm. T. Belfield, M.D 261
Correspondence:
Domestic 194, 298
Foreign, W. S. Caldwell..184, 378
Coryza, treatment of, Dr. Rudolphi 649
Croup true, pseudo-membranous laryngitis, J. A. Goldsberry, M.D 23
Curtis, Lester, M.D. The tactile hairs commonly called whiskers of certain animals 614
Cutter, Ephraim, M.D.:
A pre-embolic state 149
Hereditary taint 337
Cysts of the broad ligament of uterus, R. S. Sutton, M.D 463

### D.

Dal, J. W. M.D., cocobacteria, septica, from German of Billroth & Ephich 540
DeHart, I. N. M.D., the microscope in its relation to medicine and cerebral pathology. 242
Dental grafting. Dr. David-.... 432
Diphtheria:
Sewer gas in etiology of 513
Statistics of, Dr. E.N.Palmer.. 282
Thermic fever, œsophageal stricture, C. M. Easton, M.D. 157
Disinfectant, a new one, Dr. John Day 553
Dodge, David, M.D., placenta prævia 275
Duboisia, atropia, eserine, etc., by E. L. Holmes, M.D 355
Dudley, E. C., M.D.:
Reply to Dr. Newman on laceration of cervix 404
Puerperal laceration of cervix uteri and trachelorrhaphy. 225

### E.

Ears, objective sounds from, Dr. Holmes 49

PAGE.
Easton, C. M., M.D., diphtheria, thermic fever, œsophageal stricture.................... 157
Eczema of left auricle and auditory canal, Dr. Jones' clinics, F. C. Shaefer, M.D........... 153
Editorial:
Anent medical journals...... 202
New medical journals....... 303
The marine hospital service.. 81
Emboli (fatty) in fractures and osseous degenerations, Dr. Déjerine.................... 506
Emmet's operation, Robert Barnes, M.D.................. 643
Enuresis, nocturnal treatment of, Dr. Kelp.................. 107
Epidermis desquamation in a child born alive............. 433
Eserine, atropia, duboisia, pilocarpine, muscarine, by E. L. Holmes. M.D. .............. 355
Everett, J. T., M.D.:
Lessons from ovariotomies... 568
Studies in relation to the production of pain by weather. 253

F.

Femur:
Amputation of, by Prof. Langenbeck.................. 380
Fracture of, neck of, by E. W. Lee, M.D.................. 277
One hundred and sixty-three cases of farcture of the neck of, H. Wardner, M.D....... 4
Fistula:
Perineal, with stricture of urethra, F. E. Waxham, M.D. 46
Vesico-vaginal, puerperal vaginitis and laceration as causes of, Wm. H. Byford, M.D..................... 113
Floral tribute to Prof. Gunn... 108
Forceps, Loomis' in miscarriage, Henry A. Martin, M.D... 578
Fractures and osseous dengenerations, fatty emboli in, Dr. Déjerine.................... 506

G.

Gangrene of leg from phlegmasia dolens, R. G. Bogue, M.D. 605
Gastrostomy, Howse's operation for, C. T. Parkes ........... 636
Genu valgum, operation for.... 641
Goa powder, researches on the tree which produces, Dr. Da Silva, Lima................ 377

PAGE.
Goldspohn, A. M., M.D., cases treated by antiseptic surgery. 485
Goldsberry, J. A., M.D., true croup ..................... 23
Graham, D. W., M.D., papilloma of larynx.................. 368

H.

Hæmorrhages, intra-ventricular, producing contractions, O. C. Oliver, M.D.................. 505
Hæmorrhoids:
Cure of, by hypodermic syringe.................. 476
Treated by hypodermic syringe, discussion of Prof. Andrews' paper........... 493
Hairs, tactile, commonly called whiskers of certain animals, Dr. Lester Curtis............ 614
Hamill, R. C., M.D., opium smoking ........................ 414
Hard, Abner, M.D., case of extra uterine pregnancy........... 474
Harrington, H. L., M.D.:
Hypodermic use of morphia. 362
Spontaneous rupture of aorta. 362
Hemp smoking in Tetanus, Dr. Khostagir.................. 649
Hereditary taints, Dr. Ephraim Cutter..................... 337
Hernia, reduced by laparotomy, Dr. Terrier................. 217
Hiccough, cured by passage of œsophageal sound, Dr. E. Barré..................... 554
Hip-joint, amputation at, for osteo-sarcoma, C. T. Parkes, M.D. 502
Histology and microscopy, report on, Dr. G. F. Waters ... 627
Holmes, E. L., M.D.:
Atropia, duboisia, eserine, pilo-carpine and muscarine. 355
General miliary tuberculosis, involving choroid......... 265
Objective sounds from the ears...................... 495
Hotz, F. C., M.D.:
A case of unusually late chloroform asphyxia.......... 591
Defects of sight induced by exposure to excessive heat.. 276
Some unusual effects of atropine.................. 36
Hunt, H., M.D.. cupping in anthrax..................... 363
Hyde James Nevins, M.D., on masturbation, spermatorrhœa and impotence.............. 450

PAGE.
Hygiene of sight, Dr. Javal.... 506

I.

Index medicus, Robert Fletcher, M.D. ........................ 647
Impotence of adolescence, spermatorrhœa and masturbation, J. N. Hyde, M.D .......... .... 450
Ingals, E. Fletcher, M.D.:
How shall the degree of doctor of medicine be conferred....... ........... 409
Two cases of laryngeal tumors 369
Insane, the law as to the commitment of................. 206
Iodine, tincture, action of, on cervix uteri, Dr. Laboulbène..... 327
Items:
Alumni Association of Rush Medical College ......... 442
Appolinaris water............ 558
Attempt at blackmailing.... 333
Bulletin of public health....
California's last, best gifts... 557
Commencement exercises of Rush Medical College..... 440
Commencement exercises of Chicago Medical College.. 444
Justice to Dr. Sims.......... 109
Mr. Spencer Wells.......... 110
National catalogue of medical literature.. ............. 111
Spurious diploma........... 447

J.

Jaborandi in pleuritic effusions, Dr. Laramie................ 436
Jacket, plaster, inconvenience of, Edmund Andrews, M.D....... 358
Joints, scrofulous and tuberculous inflammation of, Dr. Schüller. ................... 105

L.

Labor, conduct of third stage of, DeLaskie Miller, M.D........ 562
Laceration and vaginitis (puerperal), as causes of vesico-vaginal fistula, Wm. H. Byford, M.D.................... ... 113
Laceration of cervix uteri, discussion of................... 365
Laparotomy, to reduce hernia, Dr. Terrier................ 217
Larynx:
Papilloma of, D. W. Graham, M.D........... ......... 368
Two cases of tumor of, E. Fletcher Ingals, M.D....... 369
Laryngitis:
Pseudo membraneous, J. A. Goldsberry, M.D........... 23
With œdema of glottis, F. E. Waxham, M.D............. 273
Lee, E. W., M.D., fracture of neck of femur.................. 277
Lefort, Prof., on antiseptic treatment of wounds............. 623
Letters:
Boston..................... 510
Philadelphia................ 516
London, Dr. C. T. Parkes.... 635
New York................. 644
Lueck, A. W., M.D., obituary notice of..................... 220
Lyman, Henry M., M.D.:
Our registration of births and deaths..... ........... 148
Translation of Prof. Lefort on antiseptic treatment of wounds ................. 623

M.

Martin, Henry A., M.D., on miscarriage and the use of Loomis' forceps............ 378
Masturbation, spermatorrhœa and impotence, J.N.Hyde, M.D. 450
Meningeal nerves, anatomy of, Dr. Duret................. 428
Metallo-therapeutics, Prof. Charcot....................... 185
Microscopy and histology, report on, Dr. G. F. Waters ... 627
Microscope in its relation to medicine and cerebral pathology, I. N. DeHart, M.D..... 242
Milk, injection of, into veins, M. Laborde................ 395
Miller, DeLaskie M.D., on conduct of third stage of labor... 562
Miscarriage, use of Loomis' forceps in, Henry A. Martin, M.D. 578
Morphia and choral in tetanus, M. Broca...... ... ....... 552
Morphia, hypodermic use of, H. L. Harrington, M.D.......... 362
Myrrh, tincture of, in whooping-cough, Dr. Campardon...... 52

N.

Nævi, cure of, C. T. Parkes, M.D., London Letter.... ........ 640
Nævus, treated by injection of cantharidine............... 555

PAGE.
Nelson, D. T., M.D., notes on subinvolution .......... 349
Nerve stretching .......... 622
Neuralgia, which electric current to use in, Dr. Rockwell. 208
Newman, W. H., M.D., on laceration of cervix uteri..........
Nipples, suberine for chapped, M. Brochard .......... 415

O.

Obituary notice of:
Dr. Frederick Andros .......... 219
Dr. A. W. Lueck, .......... 220
Dr. Lucius Clark .......... 79
Dr. John Maynard Woodworth .......... 664
Obstetrical anæsthesia, Dr. E. Hervieux .......... 99
Œsophageal stricture, thermic fever, diphtheria, C. M. Easton, M.D .......... 157
Oils, illuminating, Dr. Kedzie. 280
Oliver, O. C., M.D., reports of Society of Biology, Paris .......... 391, 394, 396, 505, 508
Opium smoking, R. C. Hamill, M.D .......... 414
Orchitis, tuberculous, treatment of, by Prof. Péan .......... 75
Osteotomy in London .......... 499
Osteo-sarcoma, amputation at hip-joint for .......... 502
Ovariotomy:
Clinical reports, service of Dr. Mary H. Thompson, reported by L. Anna Ballard, M.D .......... 600
Indications and contra-indication for, Dr. Duplay .......... 434
Ovariotomies, lessons from, J. T. Everett, M.D .......... 568

P.

Pain, studies in relation to the production of by weather, J. T. Everett, M D .......... 253
Pancreatic juice, composition of, Dr. Defresne .......... 551
Parkes, C. T., M.D., surgery in London .......... 498
London Letter .......... 635
Paralysis, general progressive treatment of, Prof. L. Meyer. 97
Pathology, transplantation of tissues, Dr. Zahn .......... 328
Periostitis mastiod, suppuration, Dr. Jones' clinic, F. C. Shaefer, M.D .......... 156

PAGE.
Phlegmasia dolens, from gangrene of leg, R. G. Bogue, M.D. 605
Piffard, H. G., M.D., tincture of thapsus .......... 200
Placenta Prævia:
David Dodge, M.D .......... 275
Discussion on .......... 167
Notes on, E. O. F. Roler, M.D. 123
John Bartlett, M.D .......... 159
Two cases of, by Mary H. Thompson, M.D .......... 267
Pleura and lung tissue, wound of, Dr. Terrier .......... 217
Pleuritic effusions, jaborandi in, Dr. Laramie .......... 431
Plumbic nitrate in skin diseases, Dr. Marsh .......... 556
Polypus and impacted cerumen in auditory meatus, Dr. Jones' clinic, J. C. Shaefer, M.D .......... 155
Practice, regulation of medical, Dr. Parker .......... 280
Pratt, G. B., M D., antiseptic surgery .......... 594
Pregnancy, extra-uterine case of, Dr. Abner Hard .......... 474
Prostate, enlargement of, Mercy Hospital reports .......... 47
Psoriasis, chrysophanic acid in. 553
Psoriasis diffusa, F. E. Waxham, M.D .......... 274

R.

Registration of births and deaths, Henry M. Lyman, M.D. 148
Report of:
Anatomical Society of Paris, O. C. Oliver, M.D .......... 397
Society of Biology of Paris, O. C. Oliver, M.D...391, 394, 396
Reviews and book notices:
Handbook of the diagnosis of skin diseases, Robert Liveing, M. D .......... 521
The antagonism of therapeutics, J. Milner Fothergill, M.D .......... 421
Atlas of diseases of membrana tympani, H. Macnaughton, M. D .......... 531
Atlas of skin diseases, L. A. Duhring, M. D .......... 521
The cell doctrine, Dr. James Tyson .......... 419
Cyclopedia of practice of medicine, Dr. H. Von Ziemssen, vol. 17 .......... 209 424
A clinical history of the medical and surgical diseases

PAGE.
of women, Robert Barnes, M. D. .... 89
Deterioration and race education .... 313
Diseases of the bladder and urethra, J. C. Skene, M. D. .. 532
Elementary quantitative analysis .... 422
Essentials of chemistry, R. A. Whitehouse .... 422
Fibrous tumors of uterus, W. H. Byford, M. D. .... 86
Guy's hospital reports, H. G. Howse, M. D. .... 211
Handbook of nursing for family and general use .... 214
Harvey and his discovery, Dr. J. M. Da Costa .... 94
Human Osteology, Dr. L. Holden .... 420
A manual of prescription writing, Dr. Matthew D. Mann .... 93
Medical and surgical electricity, Drs. Beard and Rockwell .... 528
Pareses of the sympathetic centers, Chas. T. Reber, M. D. .... 660
The pathological anatomy of the ear, H. Schwartze, M. D. 210
Physiology, preliminary course of lectures, James G. Whittaker, M. D. .... 214
Physiological therapeutics, Thos. W. Poole, M. D. .... 536
Principles and practice of gynæcology, Thos. Addis Emmet, M. D. .... 650
Rest and pain, a course of lectures, Dr. John Hilton .... 93
Sore throat, its nature, varieties and treatment, Prosser James .... 534
St. Bartholomew's Hospital reports .... 423
Surgery of the face, Francis Mason, M. D. .... 533
The throat and its diseases, Dr. Lennox Browne .... 91
A treatise on the science and practice of midwifery, W. S. Playfair, M. D. .... 535
Transactions of American Ophthalmological Society; Amercian Otological Society .... 416
Transactions of the Philadelphia pathological society, J. H. C. Simes, M. D. .... 213

PAGE.
Transactions medical society of State of Pennsylvania 307
Transactions of thirty-third annual meeting of Ohio State medical society .... 307
Rheumatism:
Acute, treatment of, by Prof. Frerichs, W. S. Caldwell, M. D. .... 293
Treatment of, in La Charité, W. S. Caldwell, M. D. .... 291
Roler, E. O. F., M. D., notes on placenta prævia, with report of fatal case .... 123
Rosemary, oil of, Drs. Kohler and Schreiber .... 329

S.

Salicylic acid, as an antaphrodisiac, Dr. Jewett .... 107
Salisbury, J. H., M. D.:
Report on chemistry .... 179
Case of cancer of rectum, in which colotomy was performed .... 272
Saturnine paralysis, alteration of the anterior roots in, M. Déjerine .... 391
Sawyer, E. W., M. D., a fatal case of inversio partialis uteri puerperalis .... 18
Scarlatina, treatment of, in La Charité, W. S. Caldwell, M. D. 285
Scrofulous and tuberculous inflammation of joints, Dr. Schüller .... 105
Sewers, intersecting of, Boston. 513
Shaefer, F. C., M. D.:
Climatology .... 136
Clinical reports of Illinois charitable eye and ear infirmary .... 153
Spermatorrhœa, masturbation and impotence, J. N. Hyde, M. D. .... 450
Stone in bladder, operation for, J. H. Brinton, M. D. .... 518
Strychnia:
Alcohol an antidote for, M. Hamean .... 626
Poisoning by, V. R. Bridges, M. D. .... 48
Subinvolution, notes on, Dr. D. T. Nelson .... 349
Surgery antiseptic, 323, 485, 501, 594
Sutton, R. S., M. D., cysts of broad ligament of uterus .... 463
Sympathetic, alterations in, of pernicious anæmia, Brigidi. 634

PAGE.
Syphilis, the poison of, Dr. Klebs ..................... 218
Syphlitic (probable) gumma in external auditory canal, Dr. Jones' clinic, F. C. Shaefer, M.D....................... 154

T.

Tetanus:
Cured by nerve stretching, Dr. Rausohoff................ 663
Cured by morphia and chloral, M. Broca................. 552
Hemp smoking in, Dr. Khastagir.................. 649
Thapsus:
Tincture of, Dr. J. I. Tucker. 52
H. G. Piffard, M. D.......... 200
Thermic fever, diphtheria œsophageal stricture, C. M. Easton, M. D.................... 157
Thompson, Mary H., M. D.:
Clinical report of cases of ovariotomy............... 600
Two cases of placenta prævia....................... 267
Tilley, R., M. D., battery hint... 519
Trachelorrhaphy, as a means of cure in puerperal laceration of cervix uteri, E. C. Dudley, M. D..................... 225
Tracheotomy:
By Prof. Langenbeck....... 389
Tumor of glottis, R. G. Bogue, M. D.................... 608
Transactions:
Of Brainard district medical society.................... 613
Of Chicago gynæcological society........53, 167, 365, 611
Of Chicago medical society, .....159, 165, 167, 276, 368, 492
Of Indiana, Illinois and Kentucky tri-State medical society...................... 66
Of Michigan State board of health................278, 615
Of Society of Biology, O. C. Oliver, M. D...........505, 508
Of State Microscopical society...................... 614
Of West Chicago medical society.............179, 277, 494
Transplantation of tissues, Dr. Zahn...... ................ 328
Tuberculosis, general miliary, involving the choroid, E. L. Holmes, M. D............... 265
Tucker, J. I., M. D., tincture of thapsus................... 52

PAGE.
Tumor:
Intra-laryngeal, R. G. Bogue, M. D.................... 375
Two cases, E. Fletcher Ingals, M.D.................... 369
Turpentine in whooping cough, Dr. Gerth.................. 322
Tympana, chronic non-suppurative inflammation of, Dr. Jones' clinic, F. C. Shaefer, M. D...................... 156
Typhoid fever, treatment of, in La Charité, W. S. Caldwell, M. D..... .................. 287

U.

Urethra, stricture of, Dr. F. E. Waxham.................45, 46
Uteri, amputation of the cervix, Prof. Langenbeck........... 385
Uterine:
Fibroid, treatment of, by Prof. Péan..................... 71
Polypi and fibroids, treatment of, by Prof. Langenbeck... 383
Utero-ovarian amputation in the Cæsarian operation, Dr. Debauge.................... 324
Uterus:
Case of partial inversion of, E. W. Sawyer, M. D....... 18
Cysts of the broad ligament of, R. S. Sutton, M. D...... 463
Discussion of subinvolution of........................ 367
Lymphatics of, Dr. Mierzjewski....... ....... ....... .... 397
Puerperal inversion of, discussion on................ 53
Subinvolution of, Dr. D. T. Nelson.................... 349

V.

Vagina, occlusion of, Prof. Péan....................... 76
Vaginitis and laceration puerperal, as causes of vesico-vaginal fistula, Wm. H. Byford, M. D....................... 113
Ventilation of buildings, Rev. Dr. Jacokes................ 279
Varicocele, radical cure of, by improved clasp, T. W. Williams, M. D................ 469
Vesico-vaginal fistula, puerperal vaginitis and laceration as causes of, Wm. H. Byford, M.D.. ..................... 113

PAGE.

### W.

Wardner, H., M. D., one hundred and sixty-three cases of fracture of neck of femur, H. Wardner, M. D. .................... 4
Waxham, F. E., M. D.:
Mercy Hospital clinical reports .................... 45
Mercy Hospital reports ..... 273
Weather, statistics in relation to the production of pain by, J. F. Everett, M. D ............. 253
Whooping cough:
Tincture of myrrh in, Dr. Campardin ............... 52
Turpentine in, Dr. Gerth .... 322
Williams, T. W., M. D., an improved clasp for radical cure of varicocele ............... 469

### Z.

Zinc, glycerine of the oxide of, Dr. Pollet .................. 44

### Books and Pamphlets Received.

A Clinical Treatise on Diseases of the Liver. By Dr. Fred. Theodore Frerichs. Translated by Chas. Murchison, M.D., F.R.C.P. Cl., pp. 228. 1879. New York: Wm. Wood & Co. Chicago: W. T. Keener.

Sixth Annual Report of State Board of Health of Michigan for Fiscal Year ending Sept. 30, 1878.

New Theory of the Great Physical Forces. By H. R. Rogers, M.D. 1878. Cl., pp. 107.

Essays in Surgical Anatomy and Surgery. By J. A. Wyeth, M.D. 1879. Cl., pp. 261. New York: Wm. Wood & Co. Chicago: Jansen, McClurg & Co.

A Practical Treatise on Surgical Diagnosis as a Manual for Practitioners and Students. By Ambrose L. Ranney, A.M., M.D. 1879. Cl., pp. 375. New York: Wm. Wood & Co. Chicago: W. T. Keener.

Chemistry; General, Medical and Pharmaceutical, including the Chemistry of the Pharmacopœia. A Manual on the Great Principles of the Sciences and their Application in Medicine and Pharmacy. By J. Anfield, M.A., PH.D. 1879. Leather, pp. 697. Philadelphia: H. C. Lea. Chicago: Jansen, McClurg & Co.

Paresis of the Sympathetic Centers from Over-Excitation by Solar Heat, Long Continued and Suddenly Withdrawn, etc. So-called Malaria; Its Etiology, Pathogenesis and Treatment. By Chas. T. Reber, M.D. 1879. Cl., pp. 112. St. Louis: Geo. Rumbold & Co.

Hints on the Obstetric Procedure. By W. B. Atkinson, A.M., M.D. Cl., pp 121. Phila.: D. G. Brinton.

A Treatise on Therapeutics, Comprising Materia Medica and Toxicology, with Special Reference to the Application of the Physiological Action of Drugs to Clinical Medicine. By H. C. Wood, Jr., M.D. Third edition, revised and enlarged. Cl., pp. 711. 1879. Phila.: J. B. Lippincott. Chicago: Jansen, McClurg & Co.

On the Diseases of the Abdomen, comprising those of the Stomach and Other Parts of the Alimentary Canal, Œsophagus, Cæcum, Intestines and Peritoneum. By S. O. Habershorn, M.D., Lond. With illustrations. Second American from Third English edition. Cl., pp. 554. 1879. Phila.: H. C. Lea. Chicago: Jansen, McClurg & Co.

Rhymes of Science: Wise and Otherwise. With illustrations. Cl., pp. 66. 1879. New York: Industrial Publication Co.

Spermatorrhœa: Its Causes, Symptoms, Results and Treatment. By Robert Bartholow, A.M., M.D. Fourth edition. Cl., pp. 178. New York: Wm. Wood & Co. Chicago: W. T. Keener.

A Guide to Therapeutics and Materia Medica. By Robert Farquharson, M.D. Second American edition. Revised by the Author. Adapted to the U. S. Pharmacopœia by F. Woodbury, M.D. Cl., pp. 498. 1879. Phila.: H. C. Lea. Chicago: Jansen, McClurg & Co.

Practical Instructions in Animal Magnetism. By J. P. F. Delouze. Translated by F. C. Hartshome. Cl., pp. 524. 1879. New York: S. R. Wells & Co.

Potts' Disease; its Pathology and Mechanical Treatment. With remarks on Rotary Lateral Curvature. By Newton M. Shaffer, M.D. Cl., pp. 82. 1879. New York: G. P. Putnam's Sons.

Demonstrations of Anatomy, being a Guide to the Knowledge of the Human Body by Dissection. By G. V. Ellis. From the 8th and revised edition. Leather, pp. 716. 1879. Phila.: H. C. Lea.

Minutes of the Medical Society of the County of New York, 1806–1878. Part I.

Transactions of the American Ophthalmological Society; 12th, 13th and 14th Annual Meetings.

Tenth Annual Report of the State Board of Health of Massachusetts. Jan., 1879.

On the Permanent Removal of Hair by Electrolysis. By Geo. H. Fox, M.D. Reprint from *Medical Record*, March 22, 1879.

The Therapeutical Society of New York. Reprint from *New York Medical Journal*, April, 1879.

The Difficulties and Dangers of Battey's Operation. By Geo. J. Englemann. Reprint from Transactions of American Medical Association.

An Address upon the Life and Character of Lunsford Pitts Yandell, M.D. By Richard O. C o wling, A.M., M.D.

National Board of Health Circular No. 3.

Yellow Fever: Its Origin and Relation to Other Malarial Fevers. By J. G. Westmoreland, M.D. Reprint from Transactions of Medical Associations of Georgia.

Iritis and Some of its Dangers. By S. J. Jones, A.M., M.D. Reprint from Transactions of Illinois State Medical Society. 1877.

Affections of the Lachrymal Apparatus. By S. J. Jones, A.M., M.D. Reprint from Ill. State Medical Society, May, 1878.

The Present State of Otology. By S. J. Jones, A.M., M.D. Rep. from Ill. State Med. Soc., 1878.

Osteotomy for Deformity of the Legs. By Chas. T. Poore. Rep. from *Med. Record*, April 26, 1879.

Inhalations in the Treatment of Pulmonary Diseases. By F. H. Davis, M.D. Reprint from *Detroit Lancet*, May, 1879.

Urethrismus or Chronic Spasmodic Stricture. By F. N. Otis, M.D. Reprint from *Hospital Gazette*, April 19, 1879.

Circulars of Information of the Bureau of Education, No. 1, 1879: Training Schools for Nurses.

Reports with Analysis on the Apollinaris Spring, Neuenahr, Rhenish Prussia, 1878.

National Board of Health Organization, etc.

Transactions of the State Medical Society of Arkansas; its Second Annual Session.

A Treatise on the Horse and Its Diseases. By J. B. Kendall, M.D.

---

TETANUS CURED BY NERVE STRETCHING. (*Cincinnati Lancet and Clinic, Jan.* 18.) Dr. Ransohoff reports the first *successful* case in this country of nerve stretching for tetanus. The patient, æt. 13, received a trivial injury to the foot from a piece of rusty iron. Tetanic symptoms appeared on the eighth day. On the twelfth day the posterior tibial nerve was well stretched, and from this time the symptoms improved and the patient was finally discharged cured. Of the five previous cases in which the operation has been resorted to for traumatic tetanus, three were by Prof. Vogt, with two successes, one by Verneuil, successful, and one partial success by Dr. Drake, of Canada.

### ANNOUNCEMENTS FOR THE MONTH.

### SOCIETY MEETINGS.

Chicago Medical Society—Mondays, June 2 and 16.

West Chicago Medical Society—Mondays, June 9 and 23.

### CLINICS.

MONDAY.

Eye and Ear Infirmary—2 p. m., Ophthalmological, by Prof. Holmes; 3 p. m., Otological, by Prof. Jones.

Mercy Hospital—1:30 p. m., Surgical, by Prof. Andrews.

Rush Medical College—2 p. m., Dermatological and Venereal, by Prof. Hyde; 3 p. m., Medical, by Dr. Bridge.

Woman's Medical College—2 p. m., Dermatological, by Dr. Maynard.

TUESDAY.

Cook County Hospital—2 to 4 p. m., Medical and Surgical Clinics.

Mercy Hospital—1:30 p. m., Medical, by Prof. Hollister.

WEDNESDAY.

Chicago Medical College—1:30 p. m., Eye and Ear, by Prof. Jones.

Rush Medical College—3:30 to 4:30 p. m., Diseases of the Chest, by Dr. E. Fletcher Ingals.

THURSDAY.

Chicago Medical College—1:30 p. m., Medical, by Prof. Quine.

Rush Medical College—3 p. m., Diseases of the Nervous System, by Prof. Lyman.

Eye and Ear Infirmary—2 p. m., Ophthalmological, by Dr. Hotz.

FRIDAY.

Cook County Hospital—2 to 4 p. m., Medical and Surgical Clinics.

Mercy Hospital—1:30 p. m., Medical, by Prof. Davis.

SATURDAY.

Rush Medical College—2 p. m., Surgical, by Prof. Gunn.

Chicago Medical College—2 p. m., Surgical, by Prof. Isham; 3 p. m., Neurological, by Prof. Jewell.

Woman's Medical College—11 a. m., Ophthalmological, by Dr. Montgomery.

Daily Clinics, from 2 to 4 p. m., at the Central Free Dispensary, and at the South Side Dispensary.

